# Identification of *Solanum lycopersicum* L. Casein Kinase I-like Gene Family and Analysis of Abiotic Stress Response

**DOI:** 10.3390/genes16070757

**Published:** 2025-06-27

**Authors:** Miao Jia, Xiaoxiao Xie, Quanhua Wang, Xiaoli Wang, Yingying Zhang

**Affiliations:** 1 College of Life Science, Shanghai Normal University, Shanghai 201400, China; jiamiao723@163.com (M.J.); wangquanhua@shnu.edu.cn (Q.W.); 2 Shanghai Key Laboratory of Protected Horticulture Technology, The Protected Horticulture Institute, Shanghai Academy of Agricultural Sciences, Shanghai 201403, China; xiaoxiaoxie@saas.sh.cn

**Keywords:** tomato (*Solanum lycopersicum*), *CKL* gene, bioinformatics analysis, abiotic stress

## Abstract

Background: Casein kinase I-like (*CKL*) protein is a member of the serine/threonine kinase *CKI* family and plays a pivotal regulatory role in various eukaryotic cellular processes, including stress responses. Objectives: This study aims to systematically identify the *CKL* gene family in the tomato genome and investigate its responsiveness to abiotic stress. Methods: Members of *SlCKL* were identified through genome-wide bioinformatics analysis, and their physicochemical properties, chromosomal localization, gene structure, conserved domains, phylogenetic relationships, cis-acting elements, cross-species collinearity, and tissue expression profiles were comprehensively analyzed. The expression patterns of *SlCKL* genes under abiotic stress were validated using real-time quantitative PCR. Results: A total of 16 *SlCKL* genes were identified and classified into three subfamilies (I–III), which are unevenly distributed across nine chromosomes, predominantly clustered at the ends. The gene structure, motifs, and functional domains exhibit high conservation. Collinearity analysis revealed stronger synteny between tomato and *Arabidopsis thaliana* or pepper compared to rice, maize, or tobacco, suggesting a common ancestral origin. The tissue expression profile indicates that *SlCKLs* are preferentially transcribed in roots. Promoter analysis and qRT-PCR validation demonstrated differential responses of *SlCKLs* to various abiotic stresses, such as drought, salt, heat, cold, and ABA treatment. Conclusions: This study represents the first systematic identification of the tomato *SlCKL* gene family, elucidating its evolutionary relationships, structural characteristics, tissue-specific expression patterns, and differential responsiveness to abiotic stress, thereby providing a critical foundation for further investigation into the molecular mechanisms underlying *CKL*-mediated abiotic stress adaptation in tomatoes.

## 1. Introduction

Casein kinase (*CK*) is a serine/threonine protein kinase that phosphorylates casein as an artificial substrate. It is ubiquitously expressed across species and tissues, playing critical roles in cell division, growth, metabolic regulation, and differentiation [1,2]. *CK* is classified into two subfamilies: casein kinase type I (*CKI*) and casein kinase type II. Notably, *CKI* exhibits high evolutionary conservation in both plants and animals [1,3]. Functionally, *CKI* operates in cytoplasmic and nuclear compartments, participating in diverse processes such as DNA repair, cell cycle control, cytokinesis, vesicle trafficking, morphogenesis, and circadian rhythm regulation [2,4,5,6,7,8]. Structurally, the *CKI* protein harbors an N-terminal kinase domain responsible for catalytic activity and a variable C-terminal regulatory domain that modulates kinase activity and subcellular localization [9,10]. Phosphorylation of the C-terminal region (extending approximately 130 amino acids beyond the catalytic domain) suppresses kinase activity, indicating its role as an autoinhibitory domain [11]. To date, preliminary investigations of *CKI* genes have been conducted in plants including rice, tobacco, and *A. thaliana*. For instance, the rice *CKI* homolog *OsCKI1* is upregulated by abscisic acid (ABA) and brassinosteroids (BR). Loss-of-function *OsCKI1* mutants display ABA/BR insensitivity, impaired primary root elongation, and reduced lateral/adventitious root formation [10]. Although plant *CKI* research lags behind animal studies, findings in *A. thaliana* provide conserved mechanistic insights at the cellular level. The *A. thaliana CKI* family has expanded to 17 members, comprising 13 casein kinase I-like (*CKL*) proteins and 4 photoregulated protein kinase (PPK) members. Functional analyses reveal that *AtCKL2* and *AtCKL3* regulate ABA-mediated seed germination and root development [12,13,14]. Promoter activity assays and GFP-tagged localization studies demonstrate functional divergence among *CKL* family members despite structural similarities, suggesting specialized roles in cellular, developmental, and physiological contexts [13]. Further mechanistic studies highlight functional specialization: *AtCKL6* organizes microtubules via its unique C-terminal domain, directly influencing cell morphology [15]; dynamic *AtCKL6*-associated vesicle-like structures translocate along actin filaments, implicating this kinase in vesicular transport [15]; *AtCKL2* stabilizes actin filaments in guard cells to modulate stomatal aperture under drought and ABA signaling, a key mechanism for gas exchange regulation [16]; *AtCKL8* regulates ethylene biosynthesis, as evidenced by dark-grown mutant phenotypes [17]. Collectively, these studies establish *CKI* as a multifunctional regulator across diverse biological pathways.

Tomato, as one of the most globally important vegetable crops, frequently encounters a wide range of abiotic stresses during cultivation. These stresses significantly hinder growth, compromise fruit quality and yield, and result in considerable economic losses. Abiotic stress refers to the adverse impacts on plant development caused by non-biological environmental factors. As a moderately salt-sensitive species, tomato exhibits dual responses to salinity: moderate salt concentrations enhance flavor, color, and soluble solid content by improving the sugar/acid ratio in fruits, whereas excessive salinity inhibits growth and compromises yield and quality [18,19]. Additionally, drought, extreme temperatures, and heavy metal stress differentially affect tomato growth, productivity, and fruit traits. Molecular studies have identified stress-responsive genes in tomatoes. For instance, Liu et al. (2012) [20] cloned two cold-tolerant genes—*shDHN* (dehydrin) and *shCHLP* (geranylgeranyl reductase)—from cold-resistant wild tomato. Overexpression of *shDHN* enhanced cold and drought resistance while promoting plant growth, whereas *shCHLP* elevated leaf chlorophyll content. Similarly, Li et al. (2013) [21] demonstrated that drought, salt, and low temperature stress induce the expression of *SpMPK1*/*SlMPK1*, a mitogen-activated protein kinase gene. Physiological analyses reveal stress-specific impacts: low temperatures reduce photosynthetic pigment content, while high temperatures increase it [22]. Stomatal conductance and photosynthetic rates initially decline but rebound during temperature stress, followed by progressive reduction under prolonged exposure [22]. Sucrose accumulation patterns in tomato fruits shift with temperature: septum sucrose levels rise between 25 and 29 °C, whereas placental and pulp sucrose decline [23]. Understanding tomato responses to salinity, drought, temperature extremes, and heavy metal exposure is essential for the development of stress-resistant cultivars and the enhancement of crop yields. However, research on the role of casein kinase I-like (*CKL*) genes in abiotic stress adaptation remains scarce, particularly in tomatoes. In this study, we conducted a systematic identification of the *CKL* gene family in tomatoes using advanced bioinformatics approaches. Comprehensive analyses encompassed protein physicochemical characteristics, chromosomal localization, conserved motifs/domains, collinearity across species, protein interaction networks, and tissue-specific expression profiles. Additionally, promoter cis-acting element analysis coupled with RT-PCR confirmed the responsiveness of *CKL* genes to multiple abiotic stressors.

## 2. Materials and Methods

### 2.1. Plant Materials and Stress Treatment

This study was conducted at the Key Laboratory of Facility Horticulture, Shanghai Academy of Agricultural Sciences. Tomato seeds of the “Micro-Tom” cultivar were provided by the Institute of Horticulture, Shanghai Academy of Agricultural Sciences.

Uniform, fully developed seeds were selected, surface-sterilized, and germinated. Seedlings were transplanted into 72-cell trays (54 cm × 28 cm) filled with a growth substrate mixture of commercial seedling substrate, peat, and vermiculite (2:1:1, *v*/*v*/*v*), with 1–2 seeds per cell. Trays were maintained in a controlled growth chamber under the following conditions: 25 °C (day)/20 °C (night), 16 h light/8 h dark photoperiod, photosynthetic photon flux density (PPFD) of 300 μmol·m^−2^·s^−1^, and relative humidity of 60–70% for 4–5 weeks.

In order to study the expression pattern of SlCKL genes under different abiotic stress conditions, tomato plants with relatively consistent growth after 30 days of culture were subjected to stress treatment. Six treatments were set up in the experiment: (1) control: tomato seedlings continued to grow normally under the environment of temperature 25 °C (day)/20 °C (night) and photoperiod 16 h day/8 h night; (2) low temperature stress: tomato seedlings were placed in an incubator with a temperature of 4 °C and other conditions unchanged for cold stress treatment; (3) high temperature stress: tomato seedlings were placed in an incubator with a temperature of 42 °C and other conditions unchanged for heat stress treatment; (4) salt stress: tomato seedlings were taken out of the soil, and the roots were directly immersed in 200 mM NaCl solution to simulate salt stress after washing the rhizosphere soil; (5) drought stress: tomato seedlings were taken out of the soil, and the roots were directly immersed in 20% PEG6000 solution to simulate drought stress after washing the rhizosphere soil; (6) heavy metal stress: tomato seedlings were taken out of the soil, and the roots were fully immersed in 400 uM CuSO4 solution after washing the rhizosphere soil. All treatments except low and high temperature stress involved seedlings being placed in incubators with temperature of 25 °C (day)/20 °C (night), photoperiod of 16 h day/8 h night, light intensity of 300 μmol ms^−1^, and relative humidity of 60–70% after treatment. Leaves were taken at 0, 6, 12, and 24 h, and immediately frozen in liquid nitrogen and stored at −80 °C for RT-PCR analysis of genes under abiotic stress. Sixteen tomato seedlings were randomly selected from each treatment, and then 6 leaves from each were mixed into a repeat, and each treatment was repeated three times.

### 2.2. RNA Extraction and Expression Analysis

Total RNA was extracted from tomato seedling leaves at different time points (0, 6, 12, 24 h) using the Bi-ospin Plant Total RNA Extraction Kit from Hangzhou Borui Technology Company (Hangzhou, China). Total RNA was used as a template for the reverse transcription of RNA in each sample. The specific method was carried out according to the HiScript II One Step RT-PCR (Novozan Biotechnology Co., Ltd., Nanjing, China) kit to obtain cDNA products. RT-PCR analysis was performed using reverse transcription cDNA as a template and eif as an internal reference gene. According to the reagent instructions of Hieffff UNICON Universal Blue qPCR SYBR Green Master Mix (Yisheng Biotechnology Co., Ltd., Shanghai, China), the reaction solutions were configured and the relevant reaction procedures were set up to calculate the expressed 2^−ΔΔCt^ value. In order to reduce the operation error, each sample was repeated three times. The primer design of each gene for RT-PCR is shown in Table 1.

### 2.3. Identification of SlCKL Gene Family Members

The Hidden Markov Model (HMM) profile of the casein kinase I-like (*CKL*) protein family was retrieved from the Pfam database (http://pfam-legacy.xfam.org/). Candidate *CKL* genes in tomato were initially identified by screening the tomato protein sequence database (downloaded from The *A. thaliana* Information Resource, TAIR: https://www.arabidopsis.org) using the HMMER 3.0 search tool. To minimize potential omissions of *SlCKL* family members due to divergence in conserved domains, the preliminary search results were further subjected to BLAST analysis against a custom-built reference library. Subsequently, a *SlCKL*-specific HMM profile was constructed and employed for a second iterative search within the tomato proteome. Redundant sequences and those with low similarity (E-value > 1 × 10^−5^) were systematically removed to ensure dataset integrity.

### 2.4. Analysis of Physicochemical Properties of SlCKL Family Genes

The isoelectric points (pI) and molecular weights of *SlCKL* proteins were predicted using the Compute pI/Mw tool on the ExPASy platform (https://web.expasy.org/compute_pi/, accessed on 21 June 2024). Protein sequence lengths were determined from annotated gene family members via TBtools based on their respective amino acid sequences.

### 2.5. Chromosomal Localization of SlCKL Genes

Chromosomal coordinates of *SlCKL* genes were mapped using the “Gene Location Visualize” module in TBtools. Genome annotation files (GTF/GFF format) and *SlCKL* gene IDs were imported to generate a visual representation of their chromosomal distribution.

### 2.6. Structural Analysis of SlCKL Genes and Proteins

Conserved domains in *SlCKL* proteins were identified using the CD-Search tool (NCBI; https://www.ncbi.nlm.nih.gov/Structure/cdd/wrpsb.cgi, accessed on 10 August 2024). Motif discovery was performed via the MEME Suite (https://meme-suite.org/) with default parameters (maximum 15 motifs, width 6–50 residues). Gene structures (exon/intron organization) and motif/domain architectures were visualized using TBtools, integrating genome-wide annotation data (GFF3 file).

### 2.7. Phylogenetic Analysis of SlCKL Genes

Protein sequences of the tomato *SlCKL* gene family members were aligned using MEGA 11.0.13, and the multiple sequence alignment results were visualized with JalView 2.11.4.0. Based on model selection analysis in MEGA, the LG+G amino acid substitution model was employed to construct a maximum likelihood phylogenetic tree with 1000 bootstrap replicates.

### 2.8. Promoter Cis-Acting Element Analysis of SlCKL Genes

Promoter regions (2000 bp upstream of the transcription start site) of *SlCKL* genes were analyzed using the PlantCARE database (https://plantcareservices.co.za/, accessed on 28 August 2024) to identify cis-regulatory elements. Predicted elements were categorized and visualized using the plotting tools in TBtools.

### 2.9. Collinearity Analysis of SlCKL Genes

Whole-genome protein sequences (FASTA format) and annotation files (GFF3 format) for pepper, *A. thaliana*, maize, rice, and tobacco were retrieved from Phytozome (https://phytozome-next.jgi.doe.gov/, accessed on 10 September 2024) and Ensembl Plants (https://plants.ensembl.org/, accessed on 11 September 2024). Syntenic relationships between tomato *SlCKL* genes and orthologs in other species were determined using MCScanX 12.0.11 and visualized through TBtools.

### 2.10. Statistical Analysis

The results of RT-PCR in this experiment were calculated by the 2^−ΔΔCt^ method. Microsoft Excel was used to summarize the data, and GraphPad Prism 8 (GraphPad Software, San Diego, CA, USA) software was used for one-way analysis of variance and the multiple comparison method (TWO-way ANOVA) to analyze the significance of different treatments. The significant difference level was set to 0.05. Finally, GraphPad Prism 8 software was used to draw the histogram and TBtools was used to draw the heat map.

## 3. Results

### 3.1. Basic Information of SlCKL Gene Family in Tomato

Through bidirectional BLAST analysis using TBtools and the NCBI online tool, we identified 16 tomato *CKL* family genes, designated as *SlCKL1* to *SlCKL16* (Table 2). The encoded proteins ranged in length from 126 to 665 amino acids (aa), with *SlCKL3* being the longest (665 aa) and *SlCKL15* the shortest (126 aa). Molecular weights (MWs) spanned 14.2 kDa to 89.32 kDa; *SlCKL15* had the lowest MW (14.2 kDa), while *SlCKL4* had the highest (89.32 kDa), indicating significant intra-family variability. Isoelectric point (pI) predictions revealed a broad range of 5.2 to 9.73. Five members (*SlCKL1/3/5/7/16*) were acidic (pI < 7), whereas the remaining eleven were alkaline. Subcellular localization predictions classified seven proteins as nuclear, five as chloroplastic, and four (*SlCKL4/5/14/16*) as extracellular, vacuolar, mitochondrial, and cytoplasmic, respectively. All members exhibited negative grand average of hydropathy (GRAVY) values (−0.82 to −0.12), confirming their hydrophilic nature. Instability index predictions ranged from 27.68 to 57.90, with nine proteins classified as unstable (index > 40) and seven as stable.

### 3.2. SlCKL Chromosome Distribution Analysis

**Figure 1:**
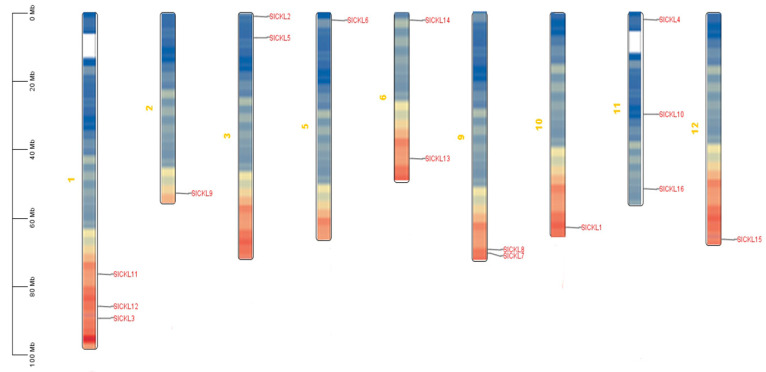
Chromosomal mapping of *SlCKL* family genes.

Chromosomal localization analysis of the *SlCKL* gene family was performed using the tomato genome sequence (Figure 1). The 16 *SlCKL* genes were distributed across nine chromosomes (1/2/3/5/6/9/10/11/12), with no members detected on chromosomes (4/7/8). Chromosomes 1 and 11 exhibited the highest density, each harboring three *SlCKL* genes. Notably, the *SlCKL* genes on chromosome 1 clustered predominantly at the distal end of the long arm (q-arm). In contrast, chromosomes (2/5/10/12) contained only a single *SlCKL* gene. With the exception of chromosome 5, where the gene localized near the proximal end of the long arm, the remaining three genes on chromosomes (2/10/12) were positioned at the distal q-arm. Collectively, the *SlCKL* gene family displayed a markedly uneven distribution across the tomato genome.

### 3.3. Structural Analysis of SlCKL Genes

**Figure 2:**
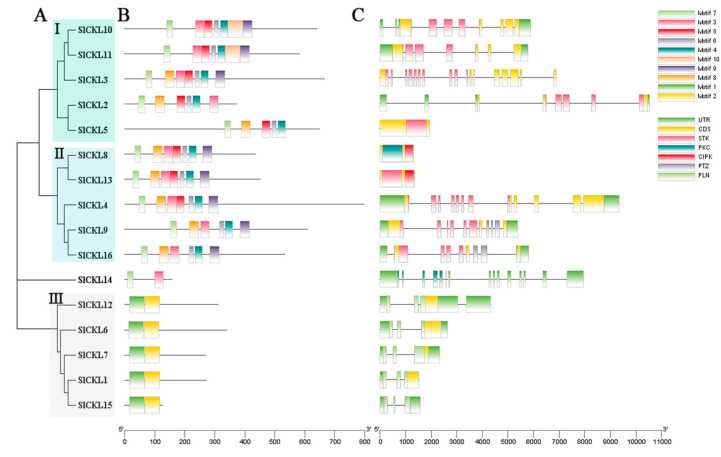
Phylogenetic tree, protein conserved domain, and gene structure of *SlCKL* family protein sequences ((**A**) used MEGA11 11.0.13 software to construct a phylogenetic tree based on the full-length sequence; (**B**) distribution of conserved motifs in *SlCKL* protein. The colored boxes represent motifs 1–10, and the scale represents 40 amino acids; (**C**) structure of the *SlCKL* gene, with a scale of 1 kb).

To investigate the functional and evolutionary characteristics of the tomato *SlCKL* gene family, we constructed a phylogenetic tree using protein sequences of all 16 members (Figure 2A). Phylogenetic analysis resolved the *SlCKL* genes into three distinct subfamilies (I–III). MEME motif analysis of the protein sequences revealed conserved structural patterns across subfamilies (Figure 2B). Subfamily I (*SlCKL2/3/5/10/11*) and subfamily II (*SlCKL4/8/9/13/16*) shared a common set of motifs (Motif3/4/5/6/7/9/10). In contrast, subfamily III (*SlCKL1/6/7/12/15*) exclusively retained Motif1 and Motif2. These findings demonstrate that motif architecture is conserved within subfamilies but divergent between them, highlighting strong evolutionary conservation within the *SlCKL* gene family.

To elucidate the structural features of *SlCKL* genes, conserved domain analysis was performed using the NCBI Conserved Domain Database (CDD; Figure 2C). All subfamily I members harbored conserved serine/threonine protein kinase domains, confirming their potential catalytic activity. Intron/exon structural analysis revealed that 13 out of 16 *SlCKL* genes (*SlCKL5/8/13* being exceptions) contained introns, suggesting evolutionary plasticity and functional diversification within the family. Subfamilies I and II exhibited higher intron density, with *SlCKL4* (11 introns), *SlCKL9* (10 introns), *SlCKL10*, and *SlCKL16* (9 introns each) showing the most complex architectures. In contrast, subfamily III members displayed minimal intronic content, typically retaining only two introns.

### 3.4. Cis-Acting Element Analysis of Promoter of SlCKL Genes

**Figure 3:**
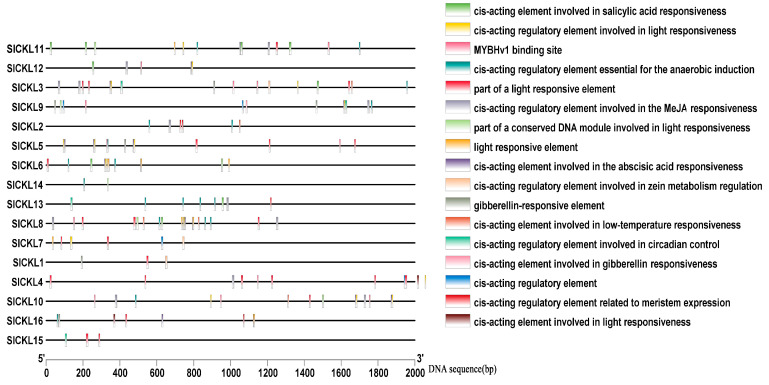
Cis-acting element of the tomato *SlCKL* promoter.

Bioinformatic analysis using the PlantCARE database revealed that the promoters of tomato *SlCKL* genes contain conserved cis-acting elements, including ubiquitous TAAT-box and CAAT-box motifs, as well as stress-responsive elements associated with light, drought, low temperature, and phytohormones (Figure 3).

*SlCKL* (*2/3/8/10*) harbor low-temperature-responsive elements (LTREs). *SlCKL* (*3/4/5/12*) possess MYB-binding sites implicated in drought responsiveness. Photoreactive elements were identified in all members except *SlCKL* (*1/2/13/14/15/16*). Anaerobic induction elements were ubiquitously distributed across *SlCKL* promoters. These findings provide a foundation for identifying transcription factors that interact with these regulatory motifs under stress conditions.

### 3.5. Multi-Species Collinearity Analysis of SlCKL Genes

**Figure 4:**
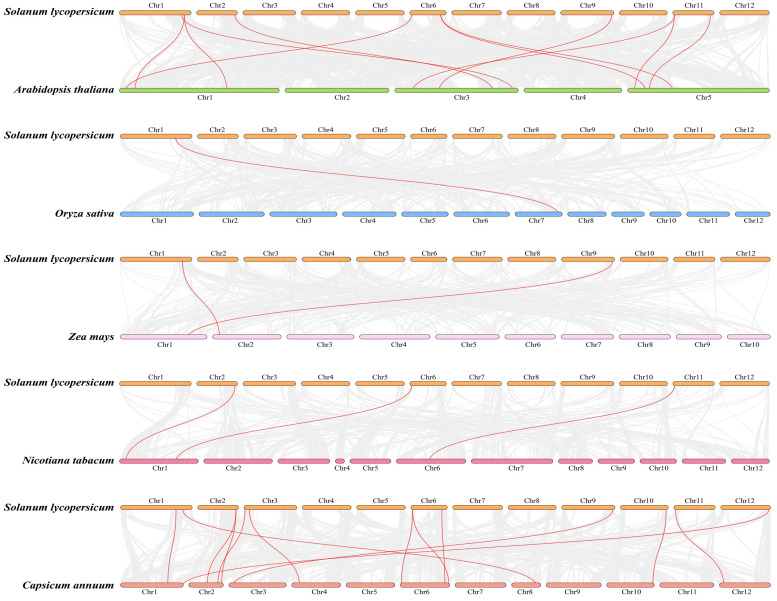
Collinearity analysis of *SlCKL* family genes between tomato and rice, maize, eggplant, wolfberry, pepper, and *A. thaliana*.

To elucidate the evolutionary history and origins of the *SlCKL* gene family, genome-wide collinearity analysis was performed between tomato and five species: *A. thaliana*, rice, maize, tobacco, and pepper (Figure 4). Comparative genomic analysis revealed distinct patterns of *CKL* gene homology between tomato and other species. Specifically, 11 homologous gene pairs were identified between tomato and *A. thaliana*, distributed across chromosomes (1/2/6/9/11). In contrast, tomato and pepper shared 13 homologous pairs localized to chromosomes (1/2/3/6/9/10/11/12). These findings suggest stronger evolutionary conservation of *CKL* genes among dicotyledonous plants. Interspecies comparisons with monocots showed limited homology: only one homologous pair between tomato and rice, two pairs with maize, and three pairs with tobacco. This sparse homology implies early divergence of *CKL* genes between tomato and monocot lineages. Collectively, the widespread presence of *CKL* homologs in both dicots and monocots, coupled with higher sequence conservation within dicots, underscores their functional importance while reflecting divergent evolutionary trajectories across plant lineages.

### 3.6. Tissue Expression Analysis of SlCKL Gene

**Figure 5:**
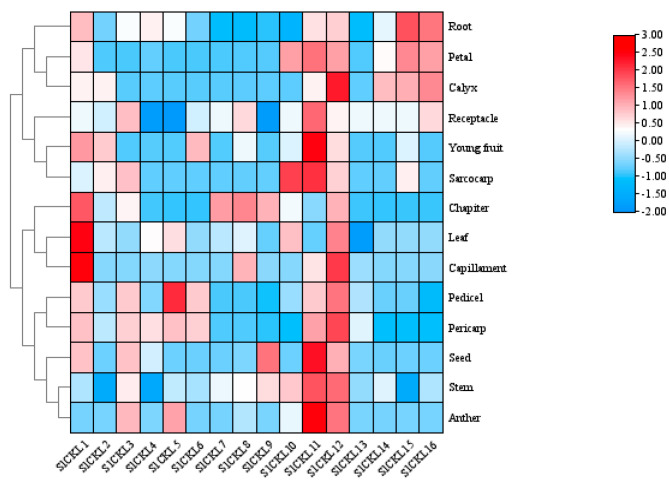
Gene expression of *SlCKL* in different organs of tomato (The corresponding color of the color band is mapped to the heat map matrix data. Near the positive color is high expression and positive correlation, while near the negative color is low expression and negative correlation).

To investigate the potential roles of *SlCKL* genes in tomato growth and development, we analyzed their expression profiles across 16 tissues at distinct developmental stages using TomExpress transcriptome data (Figure 5). The 16 *SlCKL* genes exhibited tissue-specific expression patterns with significant divergence. *SlCKL3*, *SlCKL5*, and *SlCKL11* displayed pronounced expression in roots, pedicels, and fruit pericarps. *SlCKL8*, *SlCKL13*, and *SlCKL9* showed minimal expression across all tissues. In contrast, *SlCKL4* was highly expressed in roots and pericarps, while *SlCKL16* expression was root-specific. *SlCKL12* exhibited broad expression with peak levels in pericarps and pedicels. Conversely, *SlCKL7* demonstrated low expression in all tissues, whereas *SlCKL15* was predominantly expressed in roots. These results indicate functional diversification among *SlCKL* family members in regulating tissue-specific developmental processes.

### 3.7. Expression Analysis of Tomato SlCKL Gene Under Abiotic Stress Conditions

**Figure 6:**
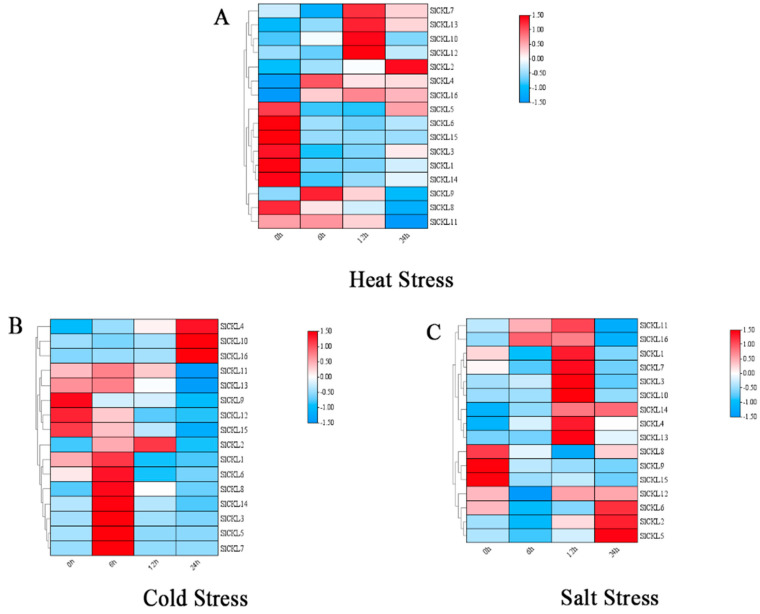

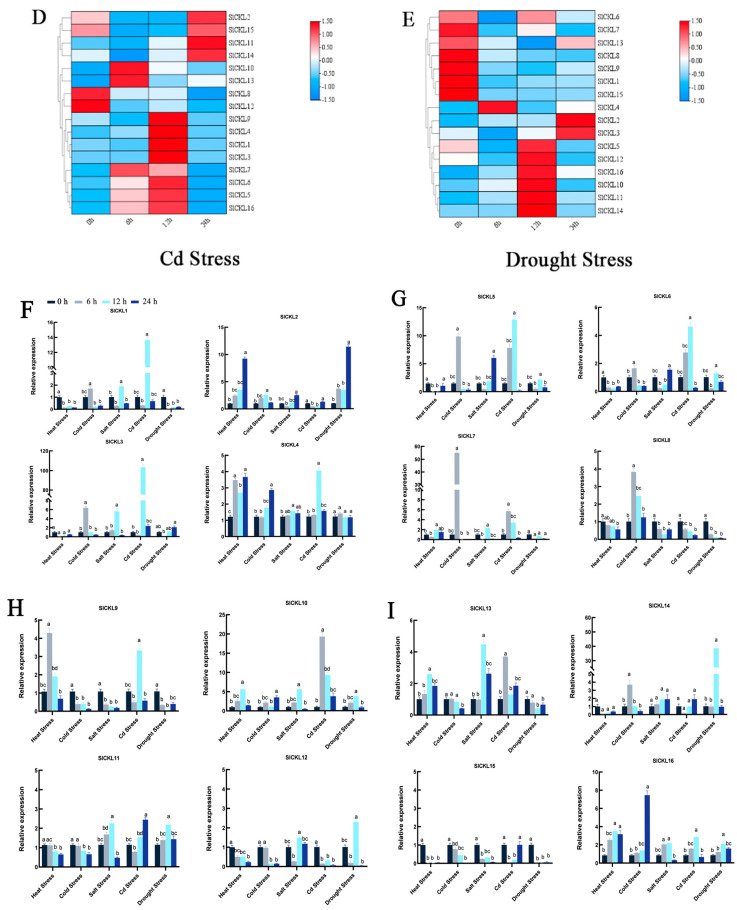
Expression of *SlCKL* in tomato seedlings after abiotic stress treatments with high temperature (42 °C), low temperature (4 °C), NaCl, CuSO4 and PE. qPCR analysis of *SlCKL* gene expression under abiotic stress treatments: (**A**) heat stress; (**B**) cold stress; (**C**) salt stress; (**D**) Cd stress; (**E**) drought stress; (**F**–**I**) expression trend of SlCKL gene between different abiotic stresses. The standard deviations are shown with error bars. Different letters indicate significant differences (*p* < 0.05).

In order to study the expression pattern of the *SlCKL* gene family under abiotic stress, we detected the expression of SlCKL in tomato seedling leaves treated with high temperature (42 °C), low temperature (4 °C), NaCl, CuSO4, and PEG abiotic stress by RT-PCR (Figure 6). The results showed that the expression trends of *SlCKL* (*2/4/9/10/16*) were similar under high temperature stress, and these genes were upregulated. However, *SlCKL* (*1/3/6/8/12/15*) showed a significant downward trend with time under high temperature stress. Under low temperature stress, the expression levels of most *SlCKL* genes showed a significant upward trend. It is worth noting that the expression levels of *SlCKL7* and *SlCKL16* increased by 58 times and 7 times under cold stress for 6 h and 24 h, respectively. The expression trend of *SlCKL* (*1/3/5/6/7/8/14*) was similar under low temperature stress, which increased significantly within 6 h of low temperature stress and decreased significantly after 6 h. Under salt stress, except *SlCKL* (*8/9/15*), which showed a downward trend, most of the other genes showed an upward trend. Under cadmium stress, except for *SlCKL* (*2/8/12/14/15*), the expression levels of other genes showed a significant upward trend. The expression levels of *SlCKL5* and *SlCKL10* under cadmium stress for 12 h reached 12 times and 19 times, respectively. The expression level of *SlCKL3* increased significantly by 106 times. Under drought stress, the expression of *SlCKL* (*2/3/10/11/12/14*) was upregulated, while *SlCKL* (*1/8/15*) was significantly decreased, and the expression of *SlCKL14* was significantly increased by 39 times under drought stress for 12 h.

## 4. Discussion

### 4.1. Identification of Tomato SlCKL Gene Family

This study presents a comprehensive bioinformatic analysis of the tomato (*Solanum lycopersicum*) casein kinase I-like (*SlCKL*) gene family. Through genome-wide identification, we identified 16 *SlCKL* genes distributed across 12 chromosomes. Phylogenetic analysis classified these genes into three distinct subfamilies (I, II, and III). Consistent with findings in A. thaliana, where 16 *AtCKL* kinases are grouped into *CKL-A*, *B*, and *C*, the *CKI* family is evolutionarily conserved across eukaryotes, playing critical roles in cell cycle regulation, development, and stress adaptation [24,25]. However, the specific functions of *CKI* in tomato remain largely unknown. We analyzed conserved domains, gene structures, and motif distributions within the *SlCKL* family. Motif distributions were largely conserved within subfamilies but divergent between them. Gene structures exhibited high conservation, consistent with prior reports [26]. Notably, the *SlCKL* family lacks intron-rich genes, suggesting potential intron loss during evolutionary diversification. Collinearity analysis between tomato and other species (*A. thaliana*, maize, pepper, tobacco, and rice) revealed stronger synteny with dicotyledons (*A. thaliana*, pepper, and tobacco) than with monocotyledons (maize and rice), supporting the closer evolutionary relationship between tomato and other dicotyledonous species [27].

Tissue-specific expression profiling of *SlCKL* genes across 16 tomato tissues (Figure 4) revealed distinct differential expression patterns. Notably, comparative studies in other species highlight functional parallels: In sesame (*Sesamum indicum*), *SeCKI* transcripts are predominantly expressed in developing seeds and show approximately threefold induction by exogenous abscisic acid (ABA). *SeCKI* regulates the *SeFAD2* promoter via phosphorylation of SebHLH transcription factors [28]. In rice (*Oryza sativa*), *OsEL1* (a casein kinase I homolog) modulates flowering time through gibberellin (GA) signaling [29], while *OsCKI1* regulates root development [10]. These findings in sesame and rice align with the observed root-predominant expression of *SlCKL* genes in tomato, suggesting potential functional conservation in root-related processes.

### 4.2. Tomato SlCKL Gene Family Responds to Different Stresses

Plants are exposed to diverse abiotic stresses, such as drought, salinity, and extreme temperatures (cold and heat). It is very important to study the function of genes in plant defense. Analysis of the promoter and gene expression patterns during growth and development and exposure to stress stimuli may help to determine their functions. Cis-acting elements are functionally categorized into three groups: growth/development-related elements (e.g., G-box), hormone-responsive elements (e.g., ABRE), and stress-responsive elements (e.g., LTR) [30,31,32]. In this study, cis-acting elements within the 2000 bp upstream promoter regions of *SlCKL* genes were analyzed. Most family members harbored motifs associated with hormonal regulation, including TATC-box-, ABA-, and SA-responsive elements, implicating *SlCKL* genes in tomato growth and abiotic stress adaptation. As shown in Figure 6, SARE (salicylic acid-responsive element) exhibited universal responsiveness across four treatments, albeit with differential expression levels among *SlCKL* paralogs. Notably, the majority of *SlCKL* genes displayed ABA-responsive motifs. Previous studies demonstrate that TATC-box [33,34] and ABA signaling [35,36] mitigate stress damage by enhancing antioxidant enzyme activity, scavenging reactive oxygen species, and accumulating osmolytes. Abscisic acid (ABA), a central mediator of plant adaptation to salinity, drought, cold, and mechanical stress, regulates developmental processes (e.g., seed dormancy) and stomatal closure under water deficit [37]. ABA synthesis is induced by multiple stressors, earning its designation as a “stress hormone” [38,39]. ABA-responsive gene expression is governed by transcription factors binding to ABRE (ABA-responsive element) motifs [40,41,42], which coordinate dehydration and salt tolerance in *A. thaliana* and rice [43]. ABRE functionality extends to abiotic stress responses in Brassica napus and other species [44,45]. We hypothesize that *SlCKL* genes enhance stress tolerance via ABA- and TATC-box-mediated pathways, though mechanistic details require further investigation. Promoter analysis identified additional cis-elements linked to cold, drought, and heat stress. Transcriptomic data corroborated *SlCKL* responsiveness to cold, drought, salinity, and heavy metals, validated by qRT-PCR. In *A. thaliana*, *CKL3* mutants exhibit downregulated ABA/stress-related genes (*ABI1, ABI4, ABI5, ABF3, KIN1, RAB18, SOS3, DREB1A*) and upregulated RD22/RD29B, revealing ABA’s need for stress-responsive gene regulation and *CKL3*’s role in early osmotic/salt stress adaptation [14]. These findings underscore the *SlCKL* family’s critical function in plant abiotic stress resistance.

## 5. Conclusions

A total of 16 *SlCKL* genes were identified in tomato, which can be divided into three families. Most genes are located in the nucleus, and the genes in the same subfamily have similarities in motif and structure. A total of 16 *SlCKL* genes were distributed on 12 chromosomes, and no *SlCKL* gene was located on chromosomes 4 and 7. The overall expression abundance of *SlCKL* gene family members was low in different tissues and under low temperature stress. It plays an important role in the growth and development of tomato roots, leaves, and peels under abiotic stress.

## Figures and Tables

**Table 1:** Primers for RT-PCR.

Gene Name	Forword (5′–3′)	Reverse (5′–3′)
*SlCKL1*	CTGGGTTGATGAGATGCAGC	GCTGCCCATTTGTTACCCAA
*SlCKL2*	CCTAGAATCGGCATGGGAGT	TGTGTGCTGCTAAAACCACC
*SlCKL3*	TTGGCGAAAGAACAGGAAGC	ACTCCTCCGTGTACTTCTGC
*SlCKL4*	GGAAAGGCCGTTTGTCTGAA	TACATAAGCGGCTGTTCCCA
*SlCKL5*	AACAGGACGAGCAAGCAAAG	GCATCACAATTCCCTGTCCC
*SlCKL6*	GCCAAGCAGAACAGATAACGA	TCCCATTCAGCCATGTGACT
*SlCKL7*	TGGGGTTAAAGAAAGGGCCA	CACATCGCAATAATCCGGCT
*SlCKL8*	AGGGTACGATGGTGCTAAGG	ATCCTCCGGGCTGATTTTGA
*SlCKL9*	GTCTCCAGCGAAACACATCC	GCCCTCTTCCAACTTCCTCT
*SlCKL10*	GAAAGGCGTGGTACTGCTTC	CGCCTTCTTGCTTCCTCTTC
*SlCKL11*	GATGACAATGCACGGCCTAG	GCCCAATCTTAGCCAGCTTC
*SlCKL12*	GACCTTGGACCCCTGAAGAA	CCTCGTATGATGTCTGGCCT
*SlCKL13*	CGAATGGGCGAAAAGGGAAA	CTTTAAGGGGAGGCCGTACA
*SlCKL14*	AGGTCGCAAAGCAAATCAGG	CTCCTGCTTTGCTCAACTCC
*SlCKL15*	TGCCTATGAATGCTGGACTTC	TCCGGCTATCAAGGACCATC
*SlCKL16*	GATGTCTGGAGTGCTGGAGT	TCCGGGTCAAGCATCTTCTT
SleiF	ATCCTTCAGAGCGGTGTTCA	ATCTCAAGAGCCTCTGGTGG

**Table 2:** Information of casein kinase I-like gene family from tomatoes.

**Gene**	**Gene Number**	**Amino** **Acids** **Length**	**Chromosomes** **Positioning**	**Pl**	**Mw**	**Subcellular** **Positioning**	**Hydrophilicity**	**Unstable** **Index**
*SlCKL1*	Solyc10g081490.2.1	271	10	6.05	30.86	nucl	−0.774	55.75
*SlCKL2*	Solyc03g006500.3.1	371	3	8.11	41.24	chlo	−0.35	39.82
*SlCKL3*	Solyc01g098980.3.1	665	1	5.2	72.68	chlo	−0.45	50.66
*SlCKL4*	Solyc11g007760.2.1	798	11	7.31	89.32	extr	−0.352	38.87
*SlCKL5*	Solyc03g043710.1.1	648	3	5.68	71.93	vacu	−0.097	27.68
*SlCKL6*	Solyc05g007710.3.1	339	5	8.60	38.65	nucl	−0.712	47.85
*SlCKL7*	Solyc09g090130.3.1	269	9	5.76	30.21	nucl	−0.751	53.54
*SlCKL8*	Solyc09g083100.1.1	435	9	8.73	48.97	chlo	−0.306	39.75
*SlCKL9*	Solyc02g090510.3.1	608	2	9.14	67.92	chlo	−0.401	52.33
*SlCKL10*	Solyc11g045610.2.1	641	11	9.03	71.16	nucl	−0.545	50.64
*SlCKL11*	Solyc01g067640.3.1	581	1	9.51	64.26	nucl	−0.657	56.96
*SlCKL12*	Solyc01g094360.3.1	310	1	7.05	34.62	nucl	−0.707	57.9
*SlCKL13*	Solyc06g068450.2.1	451	6	9.03	51.05	chlo	−0.363	35.61
*SlCKL14*	Solyc06g008320.3.1	158	6	9.21	17.94	mito	−0.207	30.8
*SlCKL15*	Solyc12g096200.2.1	126	12	9.73	14.20	nucl	−0.504	45.42
*SlCKL16*	Solyc11g065660.2.1	534	11	6.03	59.66	cyto	−0.46	37.01

## Data Availability

Data is contained within the article.

## References

[1] Tuazon P.T., Traugh J.A. (1991). Casein kinase I and II—Multipotential serine protein kinases: Structure, function, and regulation. Adv. Second. Messenger Phosphoprot. Res..

[2] Gross S.D., Anderson R.A. (1998). Casein kinase I: Spatial organization and positioning of a multifunctional protein kinase family. Cell. Signal..

[3] Knippschild U., Gocht A., Wolff S., Huber N., Löhler J., Stöter M. (2005). The casein kinase 1 family: Participation in multiple cellular processes in eukaryotes. Cell. Signal..

[4] Bidère N., Ngo V.N., Lee J., Collins C., Zheng L., Wan F., Davis R.E., Lenz G., Anderson D.E., Arnoult D. (2009). Casein kinase 1α governs antigen-receptor-induced NF-κB activation and human lymphoma cell survival. Nature.

[5] Mehra A., Shi M., Baker C.L., Colot H.V., Loros J.J., Dunlap J.C. (2009). A role for casein kinase 2 in the mechanism underlying circadian temperature compensation. Cell.

[6] Honaker Y., Piwnica-Worms H. (2010). Casein kinase 1 functions as both penultimate and ultimate kinase in regulating Cdc25A destruction. Oncogene.

[7] Rumpf C., Cipak L., Dudas A., Benko Z., Pozgajova M., Riedel C.G., Ammerer G., Mechtler K., Gregan J. (2010). Casein kinase 1 is required for efficient removal of Rec8 during meiosis I. Cell Cycle.

[8] Sugiyama Y., Hatano N., Sueyoshi N., Suetake I., Tajima S., Kinoshita E., Kinoshita-Kikuta E., Koike T., Kameshita I. (2010). The DNA-binding activity of mouse DNA methyltransferase 1 is regulated by phosphorylation with casein kinase 1δ/ε. Biochem. J..

[9] Vancura A., Sessler A., Leichus B., Kuret J. (1994). A prenylation motif is required for plasma membrane localization and biochemical function of casein kinase I in budding yeast. J. Biol. Chem..

[10] Liu W., Xu Z.H., Luo D., Xue H.W. (2003). Roles of OsCKI1, a rice casein kinase I, in root development and plant hormone sensitivity. Plant J..

[11] Graves P.R., Roach P.J. (1995). Role of COOH-terminal Phosphorylation in the Regulation of Casein Kinase Iδ (∗). J. Biol. Chem..

[12] Cui Y., Ye J., Guo X., Chang H., Yuan C., Wang Y., Hu S., Liu X., Li X. (2014). Arabidopsis casein kinase 1-like 2 involved in abscisic acid signal transduction pathways. J. Plant Interact..

[13] Lee J.Y., Taoka K., Yoo B.C., Ben-Nissan G., Kim D.J., Lucas W.J. (2005). Plasmodesmal-associated protein kinase in tobacco and Arabidopsis recognizes a subset of non-cell-autonomous proteins. Plant Cell.

[14] Wang M., Yu D., Guo X., Li X., Zhang J., Zhao L., Chang H., Hu S., Zhang C., Shi J. (2011). Casein kinase 1-Like 3 is required for abscisic acid regulation of seed germination, root growth, and gene expression in Arabidopsis. Afr. J. Biotechnol..

[15] Lee J.Y. (2009). Versatile casein kinase 1: Multiple locations and functions. Plant Signal. Behav..

[16] Zhao S., Jiang Y., Zhao Y., Huang S., Yuan M., Zhao Y., Guo Y. (2016). CASEIN KINASE1-LIKE PROTEIN2 regulates actin filament stability and stomatal closure via phosphorylation of actin depolymerizing factor. Plant Cell.

[17] Tan S.T., Xue H.W. (2014). Casein kinase 1 regulates ethylene synthesis by phosphorylating and promoting the turnover of ACS5. Cell Rep..

[18] Mizrahi Y. (1982). Effect of salinity on tomato fruit ripening. Plant Physiol..

[19] Mizrahi Y., Pasternak D.O.V. (1985). Effect of salinity on quality of various agricultural crops. Plant Soil.

[20] Liu H. (2012). Transcriptome Analysis and Related Gene Function Identification of *Solanum lycopersicum* L. Cold-Tolerant Germplasm Under Low Temperature Stress. Ph.D. Thesis.

[21] Li C., Yan J.M., Li Y.Z., Zhang Z.C., Wang Q.L., Liang Y. (2013). Silencing the SpMPK1, SpMPK2, and SpMPK3 genes in tomato reduces abscisic acid—Mediated drought tolerance. Int. J. Mol. Sci..

[22] Huanwen M., Zhihui C., Yang W. (2006). Impact of temperature stress on invertase expression and photosynthetic characteristic in tomato plant. J. Northwest Sci-Tech Univ. Agric. For..

[23] Bai P., Zou Z., Yang Z., Hu X., Zhao Y. (2010). Effects of different temperature and light treatments on sugar content in different parts of *Solanum lycopersicum* L fruit. Northwest Agric. J..

[24] Li Y., Min L., Zhang L., Hu Q., Wu Y., Li J., Xie S., Ma Y., Zhang X., Zhu L. (2018). Promoters of Arabidopsis Casein kinase I-like 2 and 7 confer specific high-temperature response in anther. Plant Mol. Biol..

[25] Wang Q. (2022). Study on Mechanism of FIM1 in *Arabidopsis thaliana* Regulating Stomatal Movement Through Dynamic Changes of Microfilaments. Ph.D. Thesis.

[26] Wang Y., Zhang J., Hu Z., Guo X., Tian S., Chen G. (2019). Genome-wide analysis of the MADS-box transcription factor family in *Solanum lycopersicum*. Int. J. Mol. Sci..

[27] Chen F., Chen Q., Lin J., Wang Y., Liu H., Liang B., Deng Y., Ren C., Zhang Y., Yang F. (2022). Identification of DIR gene family in *Solanum lycopersicum* L and analysis of its response to abiotic stress. China Agric. Sci..

[28] Kim M.J., Go Y.S., Lee S.B., Kim Y.S., Shin J.S., Min M.K., Hwang I., Suh M.C. (2010). Seed-expressed casein kinase I acts as a positive regulator of the SeFAD2 promoter via phosphorylation of the SebHLH transcription factor. Plant Mol. Biol..

[29] Dai C., Xue H.W. (2010). Rice early flowering 1, a CKI, phosphorylates DELLA protein SLR1 to negatively regulate gibberellin signalling. EMBO J..

[30] Menkens A.E., Schindler U., Cashmore A.R. (1995). The G-box: A ubiquitous regulatory DNA element in plants bound by the GBF family of bZIP proteins. Trends Biochem. Sci..

[31] Fujita Y., Fujita M., Satoh R., Maruyama K., Parvez M.M., Seki M., Hiratsu K., Ohme-Takagi M., Shinozaki K., Yamaguchi-Shinozaki K. (2005). AREB1 is a transcription activator of novel ABRE-dependent ABA signaling that enhances drought stress tolerance in Arabidopsis. Plant Cell.

[32] Liu S., Chen H., Li X., Zhang W. (2016). A low-temperature-responsive element involved in the regulation of the *Arabidopsis thaliana* At1g71850/At1g71860 divergent gene pair. Plant Cell Rep..

[33] Shariatipour N., Heidari B. (2018). Investigation of drought and salinity tolerance related genes and their regulatory mechanisms in Arabidopsis (*Arabidopsis thaliana*). Open Bioinform. J..

[34] Zhao M., Liu Z., Gan J., Yang C., Lu A., Han Q., Yang H., Xu Y., Sun G., Wu D. (2024). Identification and expression analysis of XIP gene family members in rice. Genetica.

[35] Li H.L., Sun Z.Y., Zhao L.J., Han L., Ju G.S. (2009). Effect of jasmonic acid and methyl jasmonate on the plant development and resistance. Chin. Agric. Sci. Bull..

[36] Zhu F.F., Liu Y.Q., Chen Z.X., Lan J.B. (2013). Effect of abscisic acid on physiological characteristics in *Lonicera macranthoides* seedlings under salt stress. J. Chin. Med. Mater..

[37] Tuteja N. (2007). Abscisic acid and abiotic stress signaling. Plant Signal. Behav..

[38] Mahajan S., Tuteja N. (2005). Cold, salinity and drought stresses: An overview. Arch. Biochem. Biophys..

[39] Swamy P.M., Smith B.N. (1999). Role of abscisic acid in plant stress tolerance. Curr. Sci..

[40] Thomashow M.F. (1999). Plant cold acclimation: Freezing tolerance genes and regulatory mechanisms. Annu. Rev. Plant Biol..

[41] Shinozaki K., Yamaguchi-Shinozaki K. (2000). Molecular responses to dehydration and low temperature: Differences and cross-talk between two stress signaling pathways. Curr. Opin. Plant Biol..

[42] Uno Y., Furihata T., Abe H., Yoshida R., Shinozaki K., Yamaguchi-Shinozaki K. (2000). Arabidopsis basic leucine zipper transcription factors involved in an abscisic acid-dependent signal transduction pathway under drought and high-salinity conditions. Proc. Natl. Acad. Sci. USA.

[43] Yamaguchi-Shinozaki K., Shinozaki K. (2006). Transcriptional regulatory networks in cellular responses and tolerance to dehydration and cold stresses. Annu. Rev. Plant Biol..

[44] Xu L., Lin Z., Tao Q., Liang M., Zhao G., Yin X., Fu R. (2014). Multiple NUCLEAR FACTOR Y transcription factors respond to abiotic stress in *Brassica napus* L.. PLoS ONE.

[45] Dinneny J.R., Long T.A., Wang J.Y., Jung J.W., Mace D., Pointer S., Barron C., Brady S.M., Schiefelbein J., Benfey P.N. (2008). Cell identity mediates the response of Arabidopsis roots to abiotic stress. Science.

